# The nutritional applications of garlic (*Allium sativum*) as natural feed additives in animals

**DOI:** 10.7717/peerj.11934

**Published:** 2021-08-10

**Authors:** Jiashun Chen, Fang Wang, Yexin Yin, Xiaokang Ma

**Affiliations:** College of Animal Science and Technology, Hunan Agricultural University, Changsha, Hunan, China

**Keywords:** Animal production, Garlic, Nutritional applications, Growth performance, Health

## Abstract

Garlic (*Allium sativum*) is an essential vegetable that has been widely utilized as seasoning, flavoring, culinary and in herbal remedies. Garlic contains several characteristic organosulfur compounds, such as diallyl sulfide, allicin (diallyl thiosulphate), γ-glutamylcysteine, and S-allyl cysteine (alliin) and ajoene, which garlic has beneficial effects on inflammation, oxidative stress markers, hypertension, hyperlipidaemia and endothelial function *in vitro* or in animal model. These bioactive molecules are also playing pivotal role in livestock and fisheries production apart from its application in humans. Supplementation of animal feed with garlic and its related products is consistent with the modern agricultural concept of organic animal husbandry. This review compiles the information describing the effects of feeding garlic and its extracts on selected performance parameters in animals (chicken, rabbits, ruminants, pigs and fish). This review may provide reference for scientists and entrepreneurs to investigate the applications of feeds added with garlic and allicin by-products for the improvement of animal husbandry and aquatic production.

## Introduction

The use of antibiotic growth promoters (AGPs) in feeds is considered as the greatest biotechnology of animal husbandry production in the 20th century. The AGPs have many functions, including animal performance promotion, disease prevention and treatment, which have been used in animal husbandry and aquaculture for a long time. However, the overuse of AGPs results in drug residues and bacterial resistance, which seriously affect the animal husbandry, aquatic product quality, and thus threatening food safety and human health. However, with the long term use of antibiotics, concerns have been raised regarding bacterial resistance and drug residues, increasing passage of laws banning the use of antibiotics in pig production (Bi et al., 2017). Many studies have demonstrated that natural growth promoters such as essential oil (Li et al., 2012), probiotic (Pan et al., 2017), chito-oligosaccharide (Liu et al., 2010) and other additives, can be used to replace AGPs in animal feeds without impairing the animal performance (Yang et al., 2007).

As an edible plant and one of the most well-known herbal medicines worldwide, garlic (*Allium sativum*) has generated a lot of interest throughout human history as a panacea in medicine. Phytochemical analysis showed that the main compound in garlic is allicin (diallyl thiosulphate), which is a non-protein amino acid. It is cleaved by alliinase (alliin lyase, EC 4.4.1.4, an α, β-eliminating endogenous lyase from Allium spp.) to allicin (Marchese et al., 2016), which can form many other sulfur compounds, , such as diallyl sulfide, γ-glutamyl-*S*-allyl-*L*-cysteines, and *S*-allyl-*L*-cysteine sulfoxides (alliin) and ajoene, altogether they are called allium compounds (Lawson, 1996; Li et al., 2015; Suleria et al., 2015). The main pharmacological effects of garlic are due to its featured organosulphur compounds (Tapiero, Townsend & Tew, 2004) and associated with its distinctive pungent smell and other curative properties (Macpherson et al., 2005). Moreover, researchers have found that garlic has a lot of biological functions, such as anti-microbial, anti-inflammatory, anti-atherosclerotic, anti-diabetic, anti-mutagentic, anti-carcinogenic, antioxidant and immune-modulation activities (Cullen et al., 2005; Kim, 2016; Salehia et al., 2019).

**Figure 1:**
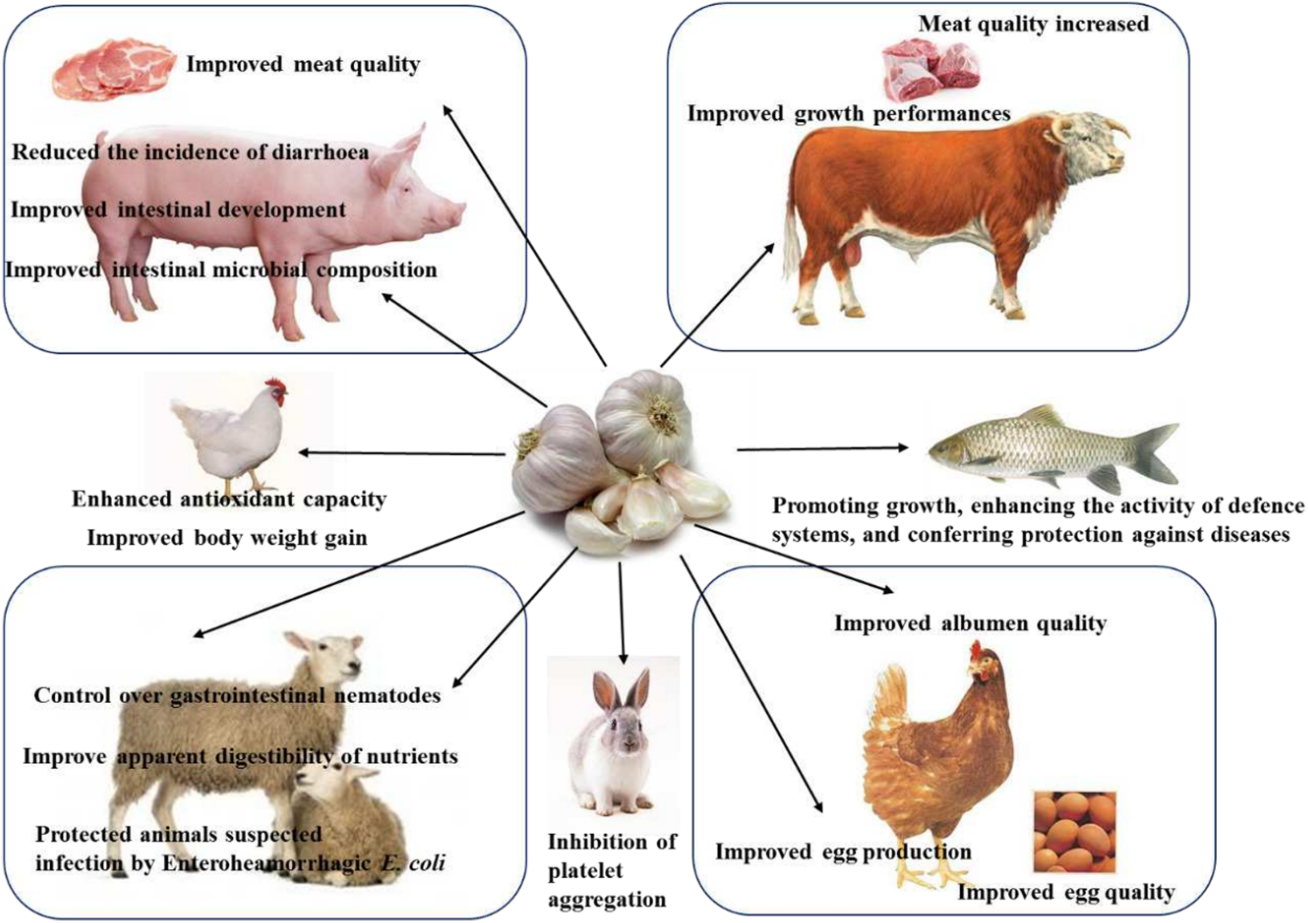
The remarkable beneficial effects of garlic in animals.

The aim of this paper, therefore, is to provide evidence for garlic and allicin to replace AGPs in animal production. This article reviews the available scientific literature on the application of garlic and its main extract allicin on livestock and aquatic animals including poultry, swine, fish, and others (Fig. 1).

## Methods

PubMed, Science Direct, CINAHL, MEDLINE, Alt Health Watch and Food Science Source electronic databases were searched online. After qualitatively evaluating the search for garlic and allicin on forums, social media, message boards, and Google, a search was made for the growth-promoting effects of garlic and allicin in livestock and aquatic animals. These keywords, “broiler”, “Laying hen”, “swine/pig/piglet”, “rabbit”, “ruminant”, “fish” were searched together with garlic. Additionally, author names and reference lists were used for further search of the selected papers for related references. Thereafter, the tone of the narrative was used to objectively evaluate the impact of garlic or allicin on animal performance and meat or egg quality.

## Nutritional applications in animals

### Effect of garlic supplementation on broiler chickens

Although dietary supplementation of garlic powder in chickens feed has been widely used, their application effects are different. Broiler fed the different levels of garlic (*Allium sativum*) powder (1.5%, 3%, and 4.5%) did not show improved body weight gain (BWG) or feed conversion ratio (FCR) (Konjufca, Pesti & Bakalli, 1997). Likewise, Sklan, Berner & Rabinowitch (1992) reported that BWG and FCR did not significantly change in chickens fed 2% garlic. Supplementation of garlic powder (1%, 3% or 5%) had no effect on growth performance (*e.g*., weight gain, feed intake, and FCR) of chickens (Choi, Park & Kim, 2010). Addition of garlic did not influence average feed intake, weight gain and FCR of chickens, but reduced abdominal fat contents (Onibi, Adebisi & Fajemisin, 2009). A study by Ao et al. (2010) revealed that fermented garlic powder increased white blood cells and lymphocyte counts, decreased cholesterol and cortisol in blood, but had no significant effect on BWG and FCR in broilers. However, Horton, Blethen & Prasad (1991) added dried garlic (0.01%, 0.1% and 1%) to broiler diets and found that average daily gain increased at the two intermediary levels of garlic (0.01% and 0.1%) during the first 21 days. A previous study also found supplementation of 1% garlic significantly improved the BWG of broilers and could be a practical alternative to antibiotics in the feeding of broiler chicken (Karangiya et al., 2016). With the 0.1 and/or 0.2% dietary administration of Korean aged garlic extract by Leukonostoc citreum SK2556, increased BWG and decreased FCR, liver weight and cecal *E. coli* count in broilers were observed (Hossain, Lee & Kim, 2014). Although supplementation of garlic-in-water neither reduced the abdominal fat deposit nor serum cholesterol, the final live weight, breast and back weights of broilers fed soyabean diet were improved by soyabean oil and garlic supplementation (Jegede, Onibi & Ogunwole, 2014). In addition, dietary supplementation of allicin has been found to enhance antioxidative capacity and reduce blood lipid level in chickens (Gong-chen et al., 2014). Elmowalid et al. (2019) also suggested promising and useful insights into dietary garlic supplementation in broilers, which may protect consumers against antibiotic residues or toxic metabolites and lower the risk of infection with bacterial pathogens. Present investigations demonstrate that supplementation of diets fed to broilers with by-products of garlic can enhance growth performance and antioxidative capacity, reduce the amount of cholesterol and cortisol in blood serum, besides the impact on FCR (Jang et al., 2018).

### Effect of garlic supplementation on laying hens

Supplementation of garlic powder (0.5% or 1%) in diets improved egg weight and reduced cholesterol content in egg yolk as well as serum triglyceride of laying hens (Yalcın et al., 2006). Olobatoke & Mulugeta (2011) suggested that 3% addition of garlic powder to layer diets efficiently improved weight and albumen quality of eggs without showing negative effects on hen performance, consumer taste or preference. Mixing layer diets with 1–3% garlic powder could improve total immunoglobulin, but yolk height, egg weight, fertility, and hatchability were not affected in White Leghorns chickens (Tesfaheywet et al., 2017; Meseret et al., 2018). A recent study conducted by Omer et al. (2019) reported that the incorporation of garlic or onion powder or the mixture of them in laying hen diets could increase shell thickness and egg weight. In contrast, Reddy, Lightsey & Maurice (1991) and Chowdhury, Chowdhury & Smith (2002) did not find garlic paste or garlic oil affect egg weight. Similarly, Hossain, Begum & Kim (2016) also reported that no change was observed in the egg weight of laying hens fed diets supplemented with aged garlic extract by *Leuconostoc mesenteroides* KCCM35046. Whereas Khan, Sardar & Anjum (2007) found that laying hens fed on 2–8% dried garlic had increased egg-production intensity along with improved egg mass. Adding 1% garlic powder into laying hen diets increased egg mass from 52 to 60 weeks of age (Abdulaziz, Khan & Rahi, 2016). The results from different studies are inconsistent, which could be explained by the differences in sources, types and the preparation methods of garlic products or powder.

On the other hand, a study conducted by Safaa (2007) reported that 2% garlic addition to the diets increased yolk weight, yolk color and Haugh unit (HU). An increasing garlic powder level in diets linearly increased the HU after two weeks of storage (Lee et al., 2014). Olobatoke & Mulugeta (2011) stated that 3% garlic powder addition in the diet induced significant increases of 0.81 mm in albumen height and 2.71 HU of fresh eggs. Mahmoud et al. (2010) indicated that oral administration of garlic liquid showed higher albumen height and HU in the laying hens. Ao et al. (2010) found that the dietary supplementation of fermented garlic powder improved yolk color, yolk height and HU, but without remarkable influence on eggshell thickness of the laying hens after 5 weeks. However, these observations were different from the findings reported by Yalcın et al. (2006), who observed that garlic did not significantly affect egg albumin, shell index, yolk index and HU values when the diets of laying hens were supplemented with garlic powder (0.5% or 1%) for 22 weeks. Kaya & Macit (2012) also found dried garlic powder did not positively influence eggshell thickness and HU in laying hens, furthermore, no change was found in the eggshell color, eggshell strength and yolk color (Hossain, Begum & Kim, 2016). The present studies cleared that inclusion of garlic powder in laying hens increased the egg weight, yolk weight, yolk color, and HU, but no effects on eggshell. Furthermore, the different garlic preparation and administration methods could be an explanation for the variations of the experimental results.

### Effect of garlic supplementation on swine

Garlic and allicin have been used to improve health and performance in livestock production (Cho, Liu & Kim, 2020). The gut development and BWG of piglet were significantly improved with the supplementation of 10 ml/100 kg body weight aged garlic extract compared with the non-garlic treatment (Tatara et al., 2005). Grela & Klebaniuk (2007) found garlic increased the BWG of piglet and decreased the incidence of piglet losses, lowered plasma triglycerides and total cholesterol concentrations. Janz et al. (2007) showed that finishing pigs preferred the garlic-treated diet by presenting significantly increased feed intake and average daily gain (ADG). Yan et al. (2011) found that fermented garlic powder supplementation (0.2%) to the basal diet increased ADG and average daily feed intake in finishing pigs after a 6-week feeding. Moreover, supplemental fermented garlic powder (0.2% or 0.4 %) increased ADG and gain/feed ratio, apparent digestibility of dry matter (DM) and nitrogen (Yan, Meng & Kim, 2012). However, Yan & Kim (2013) demonstrated that with 0.05% fermented garlic powder administration, ADG but not DM digestibility increased in weaning piglets. Huang et al. (2011) showed that allicin supplementation may improve growth performance, decrease diarrhea incidence and fly attractiveness of the faces of weanling piglets, perhaps indicating reduced dissemination of pathogens. A recent study conducted by Yun, Nyachoti & Kim (2018) found that diets supplemented with fermented garlic by *Leuconostoc mesenteroides* KCCM35046 (0.1% and 0.2%) inhibited body weight losses of lactating sows and promoted the ADG of suckling piglets in lactation period. However, the outcomes of different animal studies were not always consistent. For example, Horton, Blethen & Prasad (1991) reported that no effect was shown in the growth performance when garlic (0.1% or 1%) was added into the pig diets. Cullen et al. (2005) also found that dietary garlic administration (0.1% or 1%) reduced feed intake but improved FCR, with no significant change in ADG grower-finisher pigs. These discrepancies could happen because of the differences in type, quality of the garlic products, and animal species and age (Chen et al., 2008).

Study which focuses on the effects of garlic on meat quality of livestock was limited. Holden, Mckean & Franzenburg (1998) found garlic supplementation in diets increased pH value and lowered cooking losses of meat products from finishing pigs. Pigs fed with garlic powder (1 g/kg) showed significant improvement in meat marbling, firmness scores, pH value and water holding capacity (Chen et al., 2008). Kwon et al. (2005) and Omojola, Fagbuaro & Ayeni (2009) also reported that flavor, color, tenderness and overall acceptability were improved with garlic supplementation. In a study by Yan et al. (2011), meat marbling and the 2-thiobarbituric acid reactive substances parameters were positively influenced by fermented garlic powder (0.2% or 0.4%) supplementation.

On the other hand, the addition of garlic in swine could benefit from an improved dietary value of meat by reducing cholesterol content in muscle and the cholesterol and fat content of backfat. A previous experiment conducted by Grela et al. (2013) showed that inulin and garlic extract addition in drinking water resulted in improved carcass meatiness, including ham percentage and loin eye, reduced cholesterol content in the longissimus muscle and lowest backfat thickness of pigs. Omojola, Fagbuaro & Ayeni (2009) found increasing dietary garlic levels (0.5%, 1% and 1.5%) in pigs reduced backfat thickness and total cholesterol content in muscles. Garlic supplementation results in reduction of cholesterol in hepatocytes and triglyceride levels in blood, limitation of the formation and secretion of very low density lipoproteins, and changes in the fatty acid profile of pigs’ meat fat, which are beneficial for its dietary value (Grela et al., 2013).The combination of 0.5% garlic and 5% dandelion in diet was found most efficient in improving the growth performance, carcass quality traits, and many other parameters related with nutritional fat quality (Samolińska et al., 2020). The decline in the cholesterol content induced by garlic may be related to the reduced liver synthesis of the compound.

Moreover, Wang et al. (2011) detected that the *E. coli* count of growing pigs was largely declined by increasing amounts of fermented garlic. The effective mode of action by which allicin exhibit its antimicrobial benefits may be involved several physiological processes, which includes RNA synthesis and lipid biosynthesis (Rahman, 2007). Another potential use of allicin was investigated by Liu et al. (2018), they found that dietary 1% α-ketoglutarate combined with 0.5% allicin improved cecal microbial composition and diversity, which might further promote total volatile fatty acids metabolism in growing pigs.

These studies suggested that pigs fed diets supplemented with garlic could improve performance, meat quality and intestinal microbiota.

### Effect of garlic supplementation on rabbits

It is widely acknowledged that beneficial effects of garlic include the normalized plasma lipids, improved fibrinolytic activity, inhibited platelet aggregation, and reduced blood pressure and glucose. El-Wafa, Sedki & Ismail (2002) reported that feeding dried garlic for 8 weeks induced significant improvement in body weight, daily live weight gain of rabbit. Rabbits fed a 1% garlic powder diet exerted hypocholesterolemic and/or antiatherogenic effects and showed an inhibitory activity against cholesteryl ester transfer protein, which can delay the progression of atherosclerosis (Kwon et al., 2003). Furthermore, another study with a rabbit atherosclerosis model showed that garlic prevented the progression of the already existing atherosclerotic lesions, inhibited the occurrence of the new ones, thereby lowering the risk factor of atherosclerosis and its related cardiovascular diseases by influencing serum atherogenicity and intimal thickening (Sobenin et al., 2016). These studies demonstrated that dietary garlic could prevent the development of cholesterol-induced experimental atherosclerosis and possesses the direct anti-atherogenic activity.

### Effect of garlic supplementation on ruminants

A previous study found that supplementing garlic at 200 mg/kg of DM in a barley diet had no effects on DM intake and ruminal fermentation characteristics of lambs, neither carcass characteristics nor meat quality was affected, the fatty acid composition of back fat and liver was decreased compared to lambs fed a no-garlic diet (Chaves et al., 2008). The periodic administration of five mL garlic extract in grazing lamb decreases serum cholesterol level which can help in reducing the deposition of fat in mutton thereby improving meat quality (Amin et al., 2015). However, the use of milled garlic in commercially produced pelleted diets of sheep did not show potential effect on controlling (Strickland et al., 2009). Strickland et al. (2011) reported that the inclusion of garlic into the animal feed did not negatively affect the flavour of lamb, and the high level of garlic inclusion (3.6%) made the meat more acceptable to the panelists. Khalesizadeh et al. (2011) found that supplementation of garlic oil (0.4 g/d), monensin (0.2 g/d) and turmeric powder (20 g/d) in the lamb diets had minimal beneficial effects on total tract digestibility of lamb and did not alter feed digestion. Sheep fed with garlic stem and leaf silage diets (GS-diet, at ratio of 9:1) showed higher nitrogen and energy utilization without inhibiting ruminal fermentation (Kamruzzaman et al., 2011). Moreover, administration of commercial garlic juice product (1:1 dilution of 99.3% formula Garlic Barrier) did not alter blood packed cell volume and fecal egg counts in goats or lambs, therefore garlic is not recommended as an acute control for gastrointestinal nematodes (Burke et al., 2009).

In calf’s diets, garlic extract supplementation at 250 mg/kg body weight per day showed significantly increased feed intake, FCR, and average body weight gain, but decreased the severity of scours with lower fecal score and fecal coliform count. Ghosh et al. (2010) indicated that supplementing dairy cows with garlic (5 g/d) and juniper berry (2 g/d) essential oils increased feed digestibility in the rumen, which probably was at the expense of the flow of bypass protein to the small intestine. According to reports of Castillo-Lopez et al. (2021), dietary supplementation of garlic oil reduced the concentration of reticular total short-chain fatty acids (75.7, 71.3 and 60.1 mM) and tended to decrease ruminal acetate-to-propionate ratio (2.50, 1.78 and 1.87 ± 0.177) with no effect on ruminal pH in cattle. In addition, *in vitro* the addition of garlic juice at 0.5 mL/100 mL could enhance the production of propionate, and reduce the acetate to propionate ratio, implying that the supply of hydrogen for methanogens was limited (Kekana, Luseba & Muyu, 2021). These results suggest that garlic extract can be supplemented to the calves for better performance.

### Effect of garlic supplementation on fish

Application of garlic in fish farming has become popular for promoting growth, improving the activity of defence systems, and protecting against diseases. The bioactive compound of garlic and allicin enhanced growth by stimulating the digestive enzyme and balancing the enteric microbial flora (Talpur & Ikhwanuddin, 2012). In some other studies, dietary garlic had a beneficial effect on body weight improvement and specific growth rate in *Oreochromis niloticus* and rainbow trout (*Oncorhynchus mykiss*) (Diab, 2002; Nya & Austin, 2009; Farahi et al., 2010). Addition of 3% garlic to Nile tilapia diets improved final weight, weight gain, and specific growth rate, reduced total bacteria, and improved fish health (Shalaby, Khattab & Abdel Rahman, 2006). Moreover, dietary garlic extracts improved growth performance and feed utilization, improved dietary glucose utilization by stimulating insulin secretion, consequently improving fish body quality and feed efficiency of juvenile and fingerling Sterlet Sturgeon, *Acipenser ruthenus* (Hossain, Lee & Kim, 2014; Lee et al., 2012). Oscar fish (*Astronotus ocellatus*) fed with 10 g/kg garlic powder showed increased final weight, weight gain rate, specific growth rate, FCR, and body composition (Saghaei, Ghotbeddin & Ghatrami, 2015). In addition, the supplementation of garlic powder (1–1.5%) increased the feed utilization and the survival of red tilapia (Hossain, Lee & Kim, 2014). However, the addition of garlic and ginger (1.5%) to Nile tilapia (*O. Niloticus*) diets showed insignificant effect on growth performance, body composition, while significantly reduced lipid peroxidation and exhibited an antioxidant effect (Mahmoud et al., 2019). Therefore, addition of garlic in the diet of aquatic animal is a possible alternative rather than using synthetic or chemical supplements to improve the growth, feed utilization and survival of fish.

On the other hand, the garlic supplemented as a feed additive, increased resistance against infection of *Aeromonas hydrophila* in rainbow trout and in *Labeo rohita* (Nya & Austin, 2009; Sahu et al., 2007). Likewise, Thanikachalam, Kasi & Rathinam (2010) reported that dietary garlic peel enhanced the hematological parameters even at a low level (0.5%) supplementation and made African catfish *Clarias gariepinus* (Bloch) fingerlings highly immunopotent and more resistant to *A. hydrophila* infection. Dietary 1% garlic made Asian sea bass (*Lates calcarifer*) more resistant to the infection by *Vibrio harveyi* (Talpur & Ikhwanuddin, 2012). A recent study found that dietary 2% garlic powder increased resistance against *Yersinia ruckeri* infection in brown trout (*Salmo caspius*) through increasing the lysozyme activity (Zaefarian, Yeganeh & Adhami, 2017). In addition, Abdel-Daim, Abdelkhalek & Hassan (2015) suggested that dietary allicin attenuated deltamethrin-induced oxidative stress and might act as a therapeutic treatment to protect Nile tilapia (*Oreochromis niloticus*) on subacute deltamethrin toxicity. Administration of 0.15 mL of garlic extract per kg feed could be used to obtain optimal skin mucus immunity in female Guppy (*Poecilia reticulata*) (Motlagh et al., 2020). Thus, it has been considered that the use of dietary garlic has certainly led to protection in fish against a range of bacterial fish pathogens. There is a growing interest in studying the non-specific defence mechanism in fish, thus providing resistance to infections.

## Conclusions and future prospects

Researchers at home and abroad have conducted in-depth research on garlic and its extracts especially on their applications in animal production and human clinical studies. However, the mechanism of some of its pharmacological effects needs to be further explored. Allicin is a biologically active substance extracted from the bulb of garlic and can also be synthesized chemically. Currently, the synthetic rate of chemical allicin is 85% to 90%, whereas the extraction rate from fresh garlic is only 0.3% (Nakamoto et al., 2020). Therefore, chemically synthesized allicin has been widely used in animal production due to its low price, high purity of active ingredients and significant medicinal properties. Although it is not practical to replace antibiotics with allicin nowadays, with the development and advancement of modern science and technology, allicin and other additives will have profound potential to replace antibiotics and contribute to the production of pollution-free meat, improvement of animal welfare and sustainable development of animal husbandry. As people’s requirements for animal meat and egg quality increase, garlic and its extracts will have unique development and utilization value as green additives in improving the quality of commercial meat and eggs. Further research on garlic and its bioactive ingredients with different animal species, appropriate validation and clinical trials by exploiting the modern advances in biotechnology, nanotechnology and pharmacology will contribute to promote and propagate the nutritional and medicinal values of garlic, consequently enhance animal (chickens, laying hens, rabbits and fish) production and health.

## Additional Information and Declarations

### Data Availability

The following information was supplied regarding data availability: This is a literature review, so data is not included.
